# An Evaluation of the Repeatability of Visual Function Following Surgical Repair of Macula-Off Rhegmatogenous Retinal Detachment

**DOI:** 10.1167/tvst.12.11.35

**Published:** 2023-11-29

**Authors:** Michael J. Huvard, Jennifer L. Patnaik, David M. Kleinman, Mary Preston, David N. Zacks, Andrew J. Kocab, Jana van de Goor, Brandie D. Wagner, Steve Cho, Anne M. Lynch, Naresh Mandava

**Affiliations:** 1 Department of Ophthalmology and Visual Sciences, University of Michigan Kellogg Eye Center, Ann Arbor, MI, USA; 2 Department of Ophthalmology, University of Colorado School of Medicine, Aurora, CO, USA; 3 Flaum Eye Institute, University of Rochester Medical Center, Rochester, NY, USA; 4 ONL Therapeutics, Inc., Ann Arbor, MI, USA

**Keywords:** retinal detachment, outcome measures

## Abstract

**Purpose:**

To evaluate the reliability and reproducibility of visual function assessments for patients with macula-off rhegmatogenous retinal detachment (RRD).

**Methods:**

This prospective study included patients with unilateral macula-off RRD of <10-day duration successfully treated with a single, uncomplicated surgery at least 1 year following repair. Visual function assessments were performed at time of enrollment and 1 month later. Testing included Early Treatment Diabetic Retinopathy Study (ETDRS) best-corrected visual acuity (BCVA), low-luminance visual acuity (LLVA), low-contrast visual acuity (VA) 2.5% and 5%, contrast sensitivity assessment with Mars and Gabor patches, reading speed (acuity, speed, and critical print size), color vision testing (protan, deutan, and tritan), and microperimetry. Spectral-domain ocular coherence tomography (SD-OCT) was performed. Paired *t*-statistics were used to compare values between visits and between the study and fellow eyes.

**Results:**

Fourteen patients (9 male, 5 female) with a mean age of 69 years at time of surgery were evaluated. Correlation coefficients across the two visits were highest for ETDRS BCVA (0.97), tritan color vision testing (0.96), and low-contrast VA 5% (0.96), while the average *t*-statistic was largest for low-luminance deficit (4.2), ETDRS BCVA (4.1), and reading speed critical print size (3.7). ETDRS BCVA did not correlate with SD-OCT findings.

**Conclusions:**

ETDRS BCVA can be considered a highly reliable and reproducible outcome measure. LLVA, protan color discrimination, contrast sensitivity, and reading speed may be useful secondary outcome measures.

**Translational Relevance:**

This study provides guidance on the selection of visual function outcome measures for clinical trials of patients with macula-off RRD.

## Introduction

Rhegmatogenous retinal detachment (RRD) is a serious condition often associated with declines in visual acuity and other aspects of visual function. RRD usually requires surgery to reduce the likelihood of poor visual outcomes. Although the surgical technology employed in retinal reattachment surgery continues to advance, the general methods have remained relatively unchanged for the past 20 years. Technological innovations such as smaller incision surgery, high-speed cutters, and wide-angle viewing systems have made surgery easier for the operating surgeon, decreased procedure time, and eased postoperative patient recovery.^1^^,^^2^ However, RRD and particularly macula-off RRD remain a major cause of visual morbidity in affected patients.

Photoreceptor cell damage and death in eyes with retinal detachment is a major reason for these poor visual outcomes. Other factors such as epiretinal membranes or chronic cystoid macular edema may also contribute, but the initiation of photoreceptor apoptosis at the time of separation from the retinal pigment epithelium is a critical step in the cascade leading to permanent decreased vision.^3^^–^^5^ In a high percentage of cases, surgery restores the macular retinal microanatomy to a predetached state, but improvements in visual function to predetached levels often do not follow. Multiple large case series have documented mean final visual acuities (VAs) in macula-off RRD typically at 20/50 or worse.^6^^–^^13^ In many places in the United States, this level of acuity is poorer than that required for an unrestricted driver's license.

Although visual acuity is a key assessment for reporting results in macula-off RRD, metamorphopsia, color vision deficits, aniseikonia, problems with stereopsis, loss of contrast sensitivity, and scotomata on microperimetry have been found to varying degrees in patients following macula-off RRD.^14^^–^^28^ Similarly, advances in retinal imaging with spectral-domain ocular coherence tomography (SD-OCT) have identified changes in retinal microstructures that may predict visual recovery.^16^^,^^26^^–^^33^ Affected patients also often report subjective changes in visual function that either do not align with the extent of documented acuity decline or are in addition to such change. While procedural intervention to achieve retinal reattachment will remain for the foreseeable future as the most important method to improve long-term visual potential in eyes with macula-off RRD, and most improvement from diagnosis will likely be attributed to reattachment, there is a significant unmet need to better understand outcome measures in macula-off RRD and for new adjunctive therapies to improve visual outcomes.

This prospective study was designed to evaluate the reliability and reproducibility of visual function assessments for patients with macula-off RRD, which may aid in defining relevant endpoints for future studies or clinical trials analyzing outcomes of RRD.

## Methods

All patients included in this cross-sectional study were treated by vitreoretinal specialists at the University of Colorado (UC) Sue Anschutz-Rodgers Eye Center and were included in the UC Primary Rhegmatogenous Retinal Detachment registry. The study was approved by the Colorado Multiple Institutional Review Board and followed the tenets of the Declaration of Helsinki.

Patients with a macula-off RRD successfully repaired with a single, uncomplicated pars plana vitrectomy, with or without scleral buckle placement, and gas endotamponade who met the study's inclusion and exclusion criteria were identified from the registry and invited to participate in this study.

### Study Population

Patients who were 18 years of age or older and seen at the University of Colorado were eligible to be included in the study. All patients were required to have history of a macula-off RRD with identification of macula-off RRD status by patient history less than 7 days prior to presentation. Confirmation of macula-off status at time of diagnosis by a vitreoretinal specialist was required. The presenting visual acuity in the eye with the macula-off RRD had to be from 20/200 to hand motions, which is congruent with macula-off status.

All patients received a single, standard-of-care, retinal reattachment surgical procedure consisting of a pars plana vitrectomy (PPV), with or without scleral buckle (SB), and gas tamponade within 7 days of diagnosis of detachment. At the time of the study, all eyes were pseudophakic (routine, standard-of-care cataract extraction and intraocular lens placement in the study eye following retinal surgery was acceptable). All patients underwent surgery for retinal reattachment within the past 5 years. At the time of the study, the retina was attached for 360 degrees posterior to all barrier retinopexy. All patients were seen for their first study visit (visit 1) at least 1 year after retinal reattachment. The second study visit (visit 2) was performed approximately 30 days following visit 1.

Patients were excluded if there existed any significant ocular disease (in either eye) other than history of a macula-off RRD that would prevent the visual acuity from reaching 20/25 or better. Patients were excluded if there was evidence of intraocular inflammation, ocular or periocular infection, media opacity that would limit clinical visualization and imaging evaluation, presence of proliferative vitreoretinopathy grade C1 or worse at presentation, presence of tractional retinal detachment, or presence of any ocular or systemic condition that may have made the patient unsuitable for evaluation or assessments of the trial. Finally, patients were excluded if any postoperative complications, other than the expected cataractogenesis in phakic patients, occurred as they may impact the possibly final visual outcome.

### Study Assessments

Study assessments were performed on each eye individually with the eye that underwent RRD repair defined as the “study eye” and the eye that did not as the “fellow eye.” Assessments that did not require dilation were performed first in the following order: (1) Early Treatment Diabetic Retinopathy Study (ETDRS) best-corrected visual acuity (BCVA) (repeated once), (2) low-luminance visual acuity (LLVA) (repeated once), (3) low-contrast ETDRS BCVA 5% and 2.5%, (4) contrast sensitivity testing (repeated once) of both Mars and Metropsis grating with Gabor patches, (5) color vision testing, (6) reading speed, (7) microperimetry, (8) pupillary assessment, (9) intraocular pressure (visit 1 only), and (10) slit-lamp biomicroscopy (visit 1 only). Undilated assessments were followed by dilated procedures in the subsequent following order: (11) indirect ophthalmoscopy (visit 1 only), (12) fundus photography (visit 1 only), (13) fundus autofluorescence (visit 1 only), and (14) macular SD-OCT and OCT angiography (OCTA) (visit 1 only). Assessments that were repeated on a given visit were performed on the right and then left eye, and then the opposite with a 5-minute wait in between.

ETDRS BCVA followed standard protocol.^34^ For each eye, a routine refraction was performed. Lighthouse ETDRS Chart 1 LH 9144 (Precision Vision, Woodstock, IL, USA) was used to assess BCVA in the right eye, and then Lighthouse ETDRS Chart 2 LH 9143 (Precision Vision) was used to assess the left eye. Following a 5-minute rest period, the testing was repeated, starting with the left eye and then the right eye. Lighthouse ETDRS Chart 1 LH 9144 was always presented first. LLVA was measured by placing a 2.0 log unit neutral density filter (i.e., a filter that lowers luminance by 100 times) (Kodak Wratten filter; Kodak, Rochester, NY, USA) over the best correction for that eye and having the participant read the normally illuminated ETDRS chart. As with measuring ETDRS BCVA, the right eye was assessed followed by the left eye, and after a 5-minute rest period, the testing was repeated, starting with the left eye. A low-luminance deficit (LLD) was calculated as the difference, in logarithm of the minimum angle of resolution (logMAR) units, between the LLVA measurement and the standard VA measurement at the examination.

Low-contrast ETDRS BCVA 5% and 2.5% used a standard ETDRS measuring protocol with the respective 4 Meter Sloan Contrast Eye Test. Low contrast was performed monocularly with Sloan 5% (same chart used for each eye) and Sloan 2.5% chart (same chart used for each eye). Contrast sensitivity was tested using the Mars contrast sensitivity test.^35^ The test was performed monocularly using near correction and a testing distance of 40 to 59 cm. Uniform overhead illumination between 60 and 120 cd/m^2^ was provided, and the patient was instructed to read the chart.^36^ Testing was terminated when the patient made two consecutive errors. Mars was tested first for the right eye and then the left eye. Following a 5-minute rest period, testing was repeated, starting with the left eye. Mars chart 1 was always presented to the first eye and Mars chart 2 to the second eye. Contrast sensitivity was also tested using the Metropsis (Cambridge Research Systems Limited, Cambridge, UK) system with Gabor patches.^37^^,^^38^ Five spatial frequencies were tested monocularly at a testing distance of 150 cm using optical correction of +0.75 D or +0.50 D (whichever patient preferred) placed in front of distance correction. Patients were exposed to a brief practice test to familiarize themselves with the test and response device. Next, the patients were randomly presented the grating in a vertical or a horizontal orientation. The patient then reported the orientation of the grating. If answered correctly, the grating contrast was decreased while an incorrect response would increase the grating contrast. This adaptive procedure continued until a contrast threshold was found for each spatial frequency.

Color vision testing was assessed by the Cambridge Colour Test on the Metropsis system.^39^ The trivector test was used with a test distance of 150 cm. A clip-on add of roughly +0.75 D or +0.50 D (whichever patient preferred) was placed in front of distance correction. A C-shaped target and background were made up of many discs, each with a random luminance assigned. If the tester answered correctly, on the next presentation, the saturation of the stimulus would decrease while, if answered incorrectly, the saturation was increased. This procedure was designed to find the minimum saturation required to discriminate a target against the luminance noise.

After refraction, reading speed performance was measured through reading correction optimized at 32 cm by using the MNREAD acuity charts (Precision Vision).^40^ These charts contained 19 sentences of different print sizes ranging from 1.3 to –0.5 logMAR with each sentence containing 60 characters. The test was performed monocularly, with the right eye tested followed by the left eye, and then binocularly using charts with different sentences. MNREAD chart 1 was used for the right eye, MNREAD chart 2 for the left eye, and MNREAD chart 3 for binocular testing. The same charts were used at the next visit. The patients were instructed not to change their reading distance, and the examiner observed carefully to ensure that this was the case. Chart luminance was consistent across all visits and all subjects at ∼120 cd/m^2^. Patients were asked to read each sentence as quickly and accurately as possible, starting at the top of the chart when they hear the examiner say “start.” Each sentence was revealed one at a time, and the examiner used a stopwatch to time each sentence. The time taken to read the sentence was recorded in seconds, as was the number of words read incorrectly using a scoring sheet.

The Macular Integrity Assessment (Haag-Streit, Wedel, Germany) system was used to evaluate microperimetry to obtain the following outputs: percent reduced threshold, average threshold, fixation stability, and bivariate contour ellipse area.^41^^,^^42^ The testing program involved the 10-2 grid, 4-2 strategy.

### Ocular Coherence Tomography

Microstructural retinal imaging was performed as is standardly requested by reading centers, as advised by the Duke Reading Center on each eye with macular SD-OCT (Heidelberg Engineering, Heidelberg, Germany) at visit 1. The protocol consisted of a 97-line volume scan, 7-line high-resolution scan, enhanced depth imaging, and a custom rectangular scan set to 15 × 5 degrees with 49 B-scans. OCTA was performed in the central 6 × 6 mm (Zeiss Cirrus; Carl Zeiss Meditec, Berlin, Germany). Images were graded in a masked fashion by the Duke Reading Center. Structural factors evaluated were presence of epiretinal membrane, presence of intraretinal fluid, presence of subretinal fluid, central subfoveal thickness, average retinal thickness, boundaries of the internal limiting membrane to outer nuclear layer (ONL), boundaries of the ONL to basement membrane (BM), boundaries of the BM to external limiting membrane, total centerpoint thickness, macular volume, and the presence of any retinal anomalies (e.g., outer retinal lucency).

### Statistical Analysis

Data were entered into RedCAP, a secure web-based system. Basic descriptive statistics consisted of means, standard deviations (SDs), medians, and ranges for numerical measures. Agreement between visits was estimated using mean differences and standard deviation of differences, the paired *t*-test statistic between the two visits, and the 95% coefficients of repeatability to determine reproducibility for each ocular assessment performed on the study eyes.^43^ In addition, the Pearson correlation coefficient was used to assess correlation between visits for the study eye. The paired *t*-statistic was used to detect differences in effect size between the study and fellow eyes, with larger differences indicating that a study assessment was better at detecting differences. This was performed for each ocular assessment at each visit. SD-OCT measures between the study eye and fellow eye were compared with the signed rank test for quantitative measures and the McNemar test for categorical measures. Associations between logMAR values of ETDRS BCVA and quantitative and qualitative SD-OCT measures utilized the Spearman correlation coefficient and Wilcoxon rank sum test, respectively. SAS version 9.4 (SAS Institute, Cary, NC, USA) was used for statistical analyses.

## Results

### Demographics

A total of 14 eyes from 14 patients (9 male, 5 female) with a history of macula-off RRD in one eye were included in this study. The patient's fellow eye served as a control. Table 1 summarizes the demographic information and RRD characteristics. The mean age at onset of RRD was 69 ± 7.5 (range, 52.9–81) years. The mean duration of macular detachment prior to surgery was 4.9 ± 2.87 days (median, 5.5; range, 1–10). All patients underwent uncomplicated retinal reattachment surgery within 5 days of diagnosis (median, 2; range, 0–5). More patients underwent combined PPV/SB (8/14) compared to PPV alone (6/14).

**Table 1.** Demographic and Clinical Description of the Study Patients

Characteristic	Value
Total patients	**14**
Gender, *n* (%)	
Male	9 (64.3)
Female	5 (35.7)
Race, *n* (%)	
White^a^	12 (85.7)
African American	1 (7.1)
Other	1 (7.1)
Preoperative BCVA, *n* (%)	
20/200–20/250	2 (14.3)
20/400	2 (14.3)
Count fingers (CF)	6 (42.9)
Hand motion (HM)	4 (28.6)
Age, mean ± SD (range), y	
Time of surgery	69 ± 7.5 (52.9–81.0)
Time of first study visit	71.3 ± 7.3 (57–83)
Time from . . .	
Symptoms to diagnosis, median (range), d	1.5 (0–6)
Diagnosis to surgery, median (range), d	2 (0–5)
Symptoms to surgery, median (range), d	5.5 (1–10)
Surgery to first study visit, median (range), mo	27.9 (12.2–60.3)
Surgical technique, *n* (%)	
Pars plana vitrectomy	6 (43)
Pars plana vitrectomy/scleral buckle	8 (57)

a No patients reported Hispanic ethnicity. One patient reported white race and uncertain Hispanic ethnicity; this patient is included in white race.

### Functional Outcomes

Table 2 summarizes the functional ocular outcome measures assessed over the course of the study. Notably, across all visits, the average ETDRS BCVA was measured to be 76.15 letters (measurement 1: 76.15 ± 9.5, visit 1, 76.0 ± 9.3, visit 2; measurement 2: 76.4 ± 9.7, visit 1, 76.9 ± 9.0, visit 2), or approximately 0.17 ± 0.18 logMAR or 20/32 Snellen acuity, in study eyes while the fellow eye measured 85.65 letters (measurement 1: 85.3 ± 3.8, visit 1, 86.0 ± 3.6, visit 2; measurement 2: 85.1±3.5, visit 1, 86.2 ± 3.8, visit 2), or approximately 0.014 ± 0.07 logMAR or 20/20 Snellen acuity.^44^ A larger difference between EDTRS BCVA letters and LLVA letters was seen in the fellow eye compared to the study eye. LLD, which is defined as the difference between the standard EDTRS BCVA and LLVA, was found to be greater in fellow eyes compared to the study eye.

**Table 2.** Summary of Ocular Measures by Eye and Visit for All 14 Patients

	Study Eye (*n* = 14)		Fellow Eye (*n* = 14)	
	Visit 1	Visit 2	Visit 1	Visit 2
Outcome Measure	Mean (SD)	Mean (SD)	Mean (SD)	Mean (SD)
ETDRS BCVA 1 (letters)	75.3 (9.5)	76.0 (9.3)	85.3 (3.8)	86.0 (3.6)
ETDRS BCVA 2 (letters)	76.4 (9.7)	76.9 (9.0)	85.1 (3.5)	86.2 (3.8)
Low-luminance BCVA 1 (letters)	65.6 (9.0)	68.0 (9.3)	70.9 (5.2)	71.5 (5.5)
Low-luminance BCVA 2 (letters)	67.0 (9.2)	68.4 (8.3)	71.1 (6.5)	73.0 (5.9)
Low-luminance deficit 1 (logMAR)	−0.19 (0.09)	−0.16 (0.08)	−0.29 (0.06)	−0.28 (0.08)
Low-luminance deficit 2 (logMAR)	−0.19 (0.07)	−0.17 (0.08)	−0.28 (0.09)	−0.26 (0.08)
Sloan low-contrast BCVA 5% (letters)	60.9 (9.1)	62.9 (9.6)	66.9 (5.4)	68.4 (5.3)
Sloan low-contrast BCVA 2.5% (letters)	52.4 (9.7)	53.9 (10.2)	57.2 (5.45)	59.1 (5.87)
Mars 1 (logMAR)	1.53 (0.12)	1.53 (0.11)	1.59 (0.10)	1.57 (0.08)
Mars 2 (logMAR)	1.53 (0.10)	1.54 (0.09)	1.57 (0.09)	1.55 (0.10)
MAIA average threshold (deg^2^)	24.9 (2.6)	25.5 (1.9)	24.9 (2.4)	25.4 (1.6)
MAIA fixation stability P1 (%)	84.0 (20.4)	84.6 (21.1)	78.1 (22.4)	78.3 (26.2)
MAIA fixation stability P2 (%)	95.4 (7.3)	95.1 (11.0)	92.5 (10.3)	91.9 (14.2)
Gabor contrast sensitivity function (AUC)^a^	443.2 (468.5)		623.8 (372.2)	
Protan color vision testing^b^ (saturation)	21.2 (13.0)	20.0 (17.2)	10.7 (5.3)	13.9 (13.2)
Deutan color vision testing^b^ (saturation)	16.9 (10.4)	24.1 (27.8)	10.4 (3.1)	13.7 (9.4)
Tritan color vision testing^b^ (saturation)	44.9 (28.9)	46.5 (28.1)	35.9 (19.6)	36.8 (15.9)
Reading speed–acuity (logMAR)	0.18 (0.16)	0.17 (0.15)	0.03 (0.08)	0.07 (0.26)
Reading speed–speed (seconds)	179.4 (24.9)	184.9 (26.0)	181.1 (20.8)	190.1 (26.5)
Reading speed–critical print size (LogMAR)	0.34 (0.15)	0.29 (0.15)	0.15 (0.08)	0.12 (0.13)

AUC, area under the curve; MAIA, Macular Integrity Assessment.

a Gabor data were combined across visits.

b *n* = 12 for OD and *n* = 13 for OS at visit 1.

Table 3 lists a comparison of each of the different metrics assessed. A paired *t*-statistic value was calculated between the study eye and fellow eye for each visit. The average *t*-statistic for differences between eyes was largest (i.e., showed the most discrimination) for low-luminance deficit (mean, 4.2), ETDRS BCVA (4.1), and reading speed critical print size (3.7). Correlation coefficients across the two visits were highest for ETDRS BCVA (mean, 0.97), tritan color vision testing (0.96), and low-contrast VA 5% (0.96). The paired *t*-statistics between visits for these measures are relatively small compared to the between-eye differences, indicating similarly between the values at the two visits, although the mean differences indicate that a practice effect may be present. The *t*-statistics are unitless and can be compared across the different measures, but this statistic is a combined measure of the mean difference and the standard deviation of the difference, so a smaller value of either one can result in a small *t*-statistic. The mean difference, the standard deviation of the difference, and the coefficient of repeatability are in units similar to the measure and not comparable across measures but provide a sense of the agreement relative to clinically meaningful differences. These same statistics are also provided for the four ocular measures performed twice at visits 1 and 2 for the study eye (Table 4). Supplemental Figure S1 presents scatterplots showing the agreement within subjects for each ocular measure.

**Table 3.** Comparison of Ocular Measures at Detecting Difference between Study Eye and Fellow Eye for Each Visit and Comparing between Visit 1 and Visit 2 for Study Eye

	Comparisons Between Study Eye and Fellow Eye (Between Eye)			Comparisons Between Visit 1 and Visit 2 for Study Eye (Within Eye)			
Characteristic	Visit 1 Paired *t*-Test *t*-Statistic	Visit 2 Paired *t*-Test *t*-Statistic	Average Paired *t*-Test *t*-Statistic	Paired *t*-Test *t*-Statistic	Mean Difference	SD of Difference	Correlation Coefficient
ETDRS VA 1 letters	4.35	4.4	4.375	1.3	0.714	2.05	0.98
ETDRS VA 2 letters	3.59	4.15	3.87	0.73	0.571	2.93	0.96
Low-luminance VA 1 letters	2.25	1.56	1.905	2.7	2.36	3.27	0.96
Low-luminance VA 2 letters	1.47	2.05	1.76	1.64	1.43	3.25	0.95
Low-luminance deficit 1	4.02	4.85	4.435	3.1	0.036	0.043	0.87
Low-luminance deficit 2	3.7	4.27	3.985	2.31	0.02	0.032	0.88
Sloan low-contrast VA letters 5%	2.61	2.57	2.59	2.79	1.93	2.59	0.96
Sloan low-contrast VA letters 2.5%	1.8	2.08	1.94	1.8	1.57	3.27	0.95
Mars 1	1.97	1.99	1.98	0.13	0.003	0.082	0.78
Mars 2	1.33	0.39	0.86	0.53	0.011	0.081	0.76
MAIA average threshold	0.06	0.3	0.18	1.35	0.65	1.8	0.68
MAIA fixation stability P1	2.93	1.51	2.22	0.12	0.64	19.4	0.56
MAIA fixation stability P2	2.87	0.9	1.885	0.1	0.29	11.2	0.29
Protan color vision testing^a^	3.5	2.61	3.055	2.67	4.78	6.21	0.89
Deutan color vision testing^a^	2.42	1.4	1.91	0.58	5.24	31.4	0.12
Tritan color vision testing^a^	1.32	2.17	1.745	1.04	2.3	7.62	0.96
Reading speed–acuity	3	1.13	2.065	0.47	0.012	0.093	0.81
Reading speed–speed	0.53	0.75	0.64	1.36	7.71	18.2	0.72
Reading speed–critical print size	3.79	3.62	3.705	1.2	0.05	0.14	0.54
Gabor contrast^b^	1.17		—	—	—	—	—

a *n* = 12 for OD and *n* = 13 for OS at visit 1.

b Gabor data were combined across visits and therefore visit 1 and 2 data cannot be compared.

**Table 4.** Comparisons Within Visit for Ocular Measures Performed Twice for the Study Eye at Visits 1 and 2

Characteristic	Paired *t*-Test *t*-Statistic	Mean Difference	SD of Difference	Correlation Coefficient	95% Coefficients of Repeatability
ETDRS VA letters					
Visit 1	2.07	1.07	1.94	0.98	3.8
Visit 2	3.04	0.93	1.14	0.99	2.23
Low-luminance VA letters					
Visit 1	2.92	1.36	1.74	0.98	3.41
Visit 2	1.03	0.43	1.55	0.99	3.04
Low-luminance deficit					
Visit 1	0.59	0.006	0.036	0.91	0.071
Visit 2	0.98	0.01	0.038	0.89	0.074
Mars					
Visit 1	0	0	0.038	0.94	0.074
Visit 2	0.54	0.009	0.059	0.84	0.116

Coefficients of repeatability (CR): the difference between any two readings on the same subject is expected to be between –RC and RC for 95% of the subjects.

### Retinal Microstructural Outcomes

Microstructural imaging of the macula was obtained using SD-OCT, and the images were then graded in a masked fashion by the Duke Reading Center. Examples of images where intraretinal fluid and irregular inner retinal contour and inner retinal thinning were identified in patients with reduced VA are shown in the Figure. Quantitative and qualitative SD-OCT measures are shown for the study and fellow eyes in Table 5. ETDRS BCVA was not associated with any SD-OCT measures for either the study or fellow eyes (data not shown).

**Figure.**
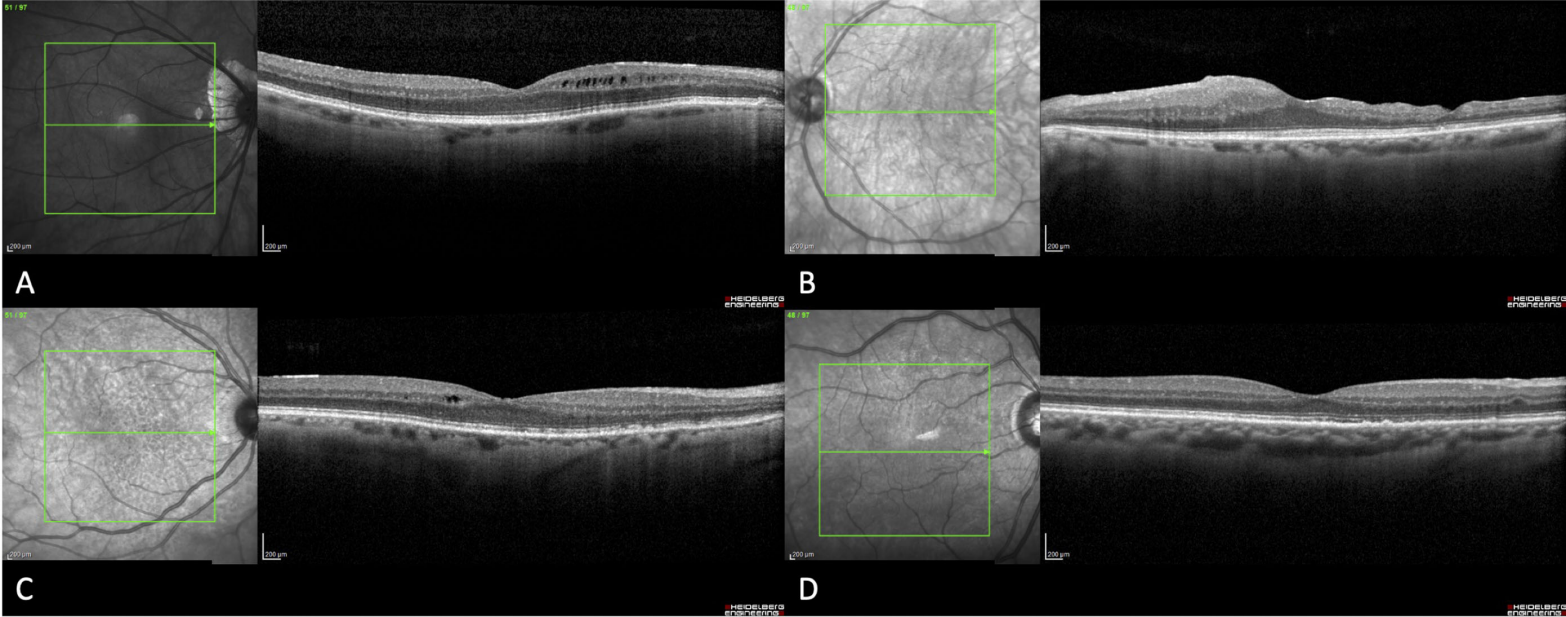
Imaging from four study patients with previously repaired macula-off rhegmatogenous retinal detachments showing varying anatomic features that do not portend to visual recovery. (**A**) SD-OCT showing mild intraretinal fluid. The best-corrected visual acuity measured Snellen equivalent 20/60. (**B**) SD-OCT showing irregular retinal contour and inner retinal thinning. Visual acuity measured Snellen equivalent of 20/70. (**C**) SD-OCT showing mild intraretinal fluid and patchy attenuation of the ellipsoid zone in an eye that measured Snellen equivalent 20/20. (**D**) SD-OCT showing mild ellipsoid zone attenuation and retinal pigment epithelial irregularity in an eye that measured Snellen equivalent 20/25.

**Table 5.** Summary of Continuous and Categorical SD-OCT Measures Comparing the Study Eye to the Fellow Eye

Outcome Measures	Study Eye (*n* = 14)	Fellow Eye (*n* = 14)	*P* Value^a^
Final visual acuity, mean (SD), logMAR	0.171 (0.183)	−0.014 (0.072)	0.0007
Continuous measures, mean (SD)			
CSVOL (mm^3^)	0.23 (0.026)	0.22 (0.02)	0.75
ELMBMT1 (µm)	54.2 (4.58)	52.93 (2.70)	0.52
ELMBMT3 (µm)	48.6 (2.79)	48.93 (2.27)	0.99
ELMBMT6 (µm)	48.86 (2.32)	50.14 (2.11)	0.18
ELMBMV1 (µm)	0.04 (0.0047)	0.04 (0.003)	0.38
ILMONLT1 (µm)	108.93 (23.85)	100.21 (17.65)	0.35
ILMONLT3 (µm)	186.14 (17.96)	180.29 (13.56)	0.03
ILMONLT6 (µm)	165.36 (15.71)	155.57 (11.93)	0.001
ILMONLV1 (µm)	0.09 (0.02)	0.079 (0.02)	0.43
ONLBMT1 (µm)	150.29 (13.23)	150.36 (8.05)	0.97
ONLBMT3 (µm)	118.9 (7.09)	120.5 (7.79)	0.15
ONLBMT6 (µm)	108.3 (6.90)	109 (6.95)	0.49
ONLBMV1 (µm)	0.12 (0.01)	0.12 (0.01)	0.99
Average 1-mm thickness (mm^3^)	290.5 (31.1)	283.14 (20.20)	0.68
Average 3-mm thickness (mm^3^)	335.07 (16.71)	332.57 (13.56)	0.37
Average 6-mm thickness (mm^3^)	302.2 (15.99)	294 (12.63)	0.007
Macular volume (mm^3^)	8.54 (0.45)	8.31 (0.35)	0.008
Total centerpoint thickness (mm^3^)	224.14 (48.51)	228.5 (23.87)	0.53
Categorical measures (*n*)			
Presence of IRF	4	0	^b^
Presence of ERM	7	3	0.05
Presence of ANO	4	6	0.32

ANO, anomaly not otherwise specified; CSVOL, central subfield volume; ELMBMT, external limiting membrane to base membrane; ERM, epiretinal membrane; ILMONL, internal limiting membrane to outer nuclear layer; IRF, intraretinal fluid; ONLBMT, outer nuclear layer to basement membrane.

a Signed rank test used for qualitative measures and McNemar's for categorical measures.

b Not calculable.

## Discussion

This study evaluated multiple ocular outcome measures to determine which metrics are most capable of reliably detecting a decline in function after macula-off RRD. Of all the outcome measures tested, ETDRS BCVA best fit the criteria of being able to detect a difference between the study eye and fellow eye (high *t*-statistic) and being highly correlated over time to the within eye Pearson correlation coefficient. Contrast sensitivity, color vision testing, microperimetry, reading speed–acuity, and reading speed–speed were found to be less able to detect differences between the study and fellow eyes. Interestingly, microperimetry was the least sensitive measure in detecting a difference in visual function between the study and fellow eye. Although other studies of RRDs scrutinize which outcomes are most impacted by RRD and their relation to visual recovery, there is a paucity of literature validating the accuracy or reproducibility of these metrics.

Early series on RRD outcomes focused on BCVA as the primary endpoint. In our study population, the average final postoperative BCVA was approximately 20/32 (Snellen equivalent of 75 to77 ETDRS letters read), which is consistent with select reports in the medical literature describing results in patients with a shorter duration of macular detachment (median, 5.5 days).^7^^–^^9^^,^^12^ It has been shown that BCVA may improve for up to 1 year after retinal reattachment, and the fact that this study assessed all patients more than 1 year after successful surgery suggests these are optimal results.^13^ Visual outcomes after retinal reattachment surgery have not improved substantially since the first studies reporting on these findings were published.^6^ Furthermore, even patients who have better than average BCVA after macula-off RRD report that the quality of their vision remains noticeably poorer than the fellow eye.^21^

Contrast sensitivity has emerged as an important component of visual function and has been found to correlate with real-world activities.^45^ Numerous studies have reported a decrease in contrast sensitivity following macula-off RRD and a strong correlation between decreased contrast sensitivity and vision-related quality of life (VR-QOL).^18^^,^^19^^,^^22^^,^^25^ One study reported reduced contrast sensitivity following RRD repair correlated with VR-QOL while VA did not.^22^ Contrast sensitivity has also been found to be subnormal in patients with macula-on RRD repair who otherwise have normal visual acuity.^23^ Recently, a novel computer contrast sensitivity test has been shown to be highly sensitive and precise at detecting small differences in contrast sensitivity function, perhaps making contrast sensitivity an important visual function endpoint for clinical trial design.^45^ However, compared to ETDRS BCVA, we found that assessments of contrast sensitivity were less effective or reliable at identifying and detecting a difference between the study eye and fellow eye. We assessed contrast sensitivity with low-contrast VA, Mars letter contrast sensitivity test, and Gabor patches. To our knowledge, Gabor patches have been used to study contrast sensitivity in settings of glaucoma and central serous chorioretinopathy but not RRD.^38^^,^^46^ Gabor patches offer the ability to assess contrast sensitivity over a large range of spatial frequencies, including precise measurements relative to the contrast sensitivity function peak and cutoff frequency of the central or peripheral visual field, which provides more information than traditional Pelli–Robson or Mars testing. Interestingly, we found that there was no statistically significant difference measured in the study eye versus fellow eye with this metric. Low-contrast VA and Mars were moderately effective at detecting a difference between the study eye and fellow eye, although the former was more reproducible. Despite these findings, contrast sensitivity may still be a useful secondary clinical endpoint when interpreting visual function after RRD repair.^47^

Changes in color vision have been previously reported in eyes after RRD.^16^^,^^18^^,^^19^ We found that protan color discrimination, compared to deutan or tritan color discrimination assessments, was best able to detect a difference between the study eye and fellow eye. Although less sensitive and reliable than ETDRS BCVA, color vision discrimination—and protan color discrimination in particular—may similarly be an informative secondary endpoint for evaluating outcomes in RRD.

We were unable to identify a study in the peer-reviewed medical literature that assessed LLVA and reading speed in the setting of macula-off RRD, although there are reports on LLVA in the setting of age-related macular degeneration, central serous chorioretinopathy, choroideremia, and other inherited retinal diseases.^48^^–^^51^ We found that LLVA, although highly reproducible, was less able to detect a difference compared to ETDRS BCVA in this patient population. LLD, which has the highest average *t*-statistic, is a metric calculated by the difference between ETDRS BCVA and LLVA. Therefore, LLD cannot stand alone as an outcome measure because it is cofounded by ETDRS BCVA. This population showed a relatively larger difference between ETDRS BCVA and LLVA (and therefore LLD) in the fellow eyes compared to the study eyes. This finding may indicate that there is a relative ceiling of LLVA as it has been shown that LLVA declines with age, more than standard VA, thereby resulting in a larger LLD.^52^^,^^53^ More research is needed to fully understand the normal range of LLD in healthy participants across different demographics.

Reading speed has similarly been studied in the context of age-related macular degeneration among other conditions, but there is scant evidence of its use in postoperative RRD patients.^49^ In our study, reading speed–critical print size was able to detect a difference between the study eye and fellow eye, but it was unreliable. The other components of reading speed—speed and acuity—were less able to detect differences and even less reliable.

Characterization of retinal microstructures with SD-OCT and other advanced imaging has emerged as an important method in assessing preoperative and postoperative factors that may predict visual recovery after RRD. The most commonly cited postoperative findings that correlate with visual recovery are integrities of the external limiting membrane and the photoreceptor inner segment/outer segment junction.^26^^,^^30^^,^^32^^,^^54^^,^^55^ This study suggests retinal microstructure parameters by SD-OCT imaging do not appear to be a particularly useful endpoint in this patient population. Although there were several continuous SD-OCT measures that reached a statistically significant difference between the study eye and fellow eye, there was no association of these measures between the final visual outcome for either the study eye or fellow eye. This lack of association to BCVA suggests SD-OCT has unclear clinical implications in certain cases or that our sample size was not large enough to detect significant associations. The Figure demonstrates several eyes with various findings that do not correlate to final visual outcome. Others have reported that changes in retinal structures after macula-off RRD on SD-OCT do not correlate with visually significant metamorphopsia.^16^ Recent use of adaptive optics SD-OCT to assess photoreceptors in a 1-year period after macula-off RRD shows that cone morphology and function significantly improve in the year following retinal reattachment, but functional impairment remains.^28^ Further research is needed to validate the use of these metrics.

This study has several limitations. First, the sample size is small. Without knowing the effect size of the intervention, it is not possible to perform a power calculation. However, given our findings, the presented data can be used to help support a power calculation for future studies. Second, macula-off RRD with a duration of macular detachment of more than 7 days was not included, and there may have been selection bias in those who opted to return to the study center and participate in this study. Nevertheless, this evaluation benefits from its utilization of multiple assessments of visual function often with test–retest validations. SD-OCT was performed as well. Furthermore, patients in this study were examined at least 1 year after repair, when retinal recovery has most likely stabilized. Third, the study population represents a limited subset of patients, all pseudophakic at the time of the study, who had macula-off RRD repaired with a single surgical intervention (PPV with or without SB). Retinal detachments, their clinical course, and their methods of repair are heterogenous. Additional investigation is needed to expand the overall applicability of our study, but the data presented represent an important starting point. In addition, binocular visual functional assessments were not possible due to the study design, but they are a meaningful aspect of a patient's visual experience. Finally, ocular measurements were performed up to four times over the two visits, and there may be subject memory recall as indicated by the slight improvement in some measures over time.

In conclusion, we describe a significant effort to validate the effectiveness and reliability of multiple measures of visual function after macula-off RRD repair. ETDRS BCVA was found to be the outcome measure most capable of reliably detecting a difference between the study eye and fellow eye. Others metrics, such as LLVA, protan color discrimination, contrast sensitivity, and reading speed, may be useful as secondary outcome measures. Importantly, this study made use of eligibility criteria similar to what would likely be seen in a prospective trial assessing the efficacy of an adjunctive therapeutic for improving postoperative visual function in macula-off RRD. The repeatability of ETDRS BCVA and its associated clear decline compared to the untreated fellow eye confirm the importance of visual acuity testing as a key assessment in future registrational studies of visual outcomes in macula-off RRD.

## Supplementary Material

Supplement 1
