# Supplementary material for: An Evaluation of the Repeatability of Visual Function Following Surgical Repair of Macula-Off Rhegmatogenous Retinal Detachment
Source: Transl Vis Sci Technol. 2023 Nov 29;12(11):35. doi: 10.1167/tvst.12.11.35 (PMC10691393; doi:10.1167/tvst.12.11.35)
Supplement: Supplement 1 [file tvst-12-11-35_s001.pdf]

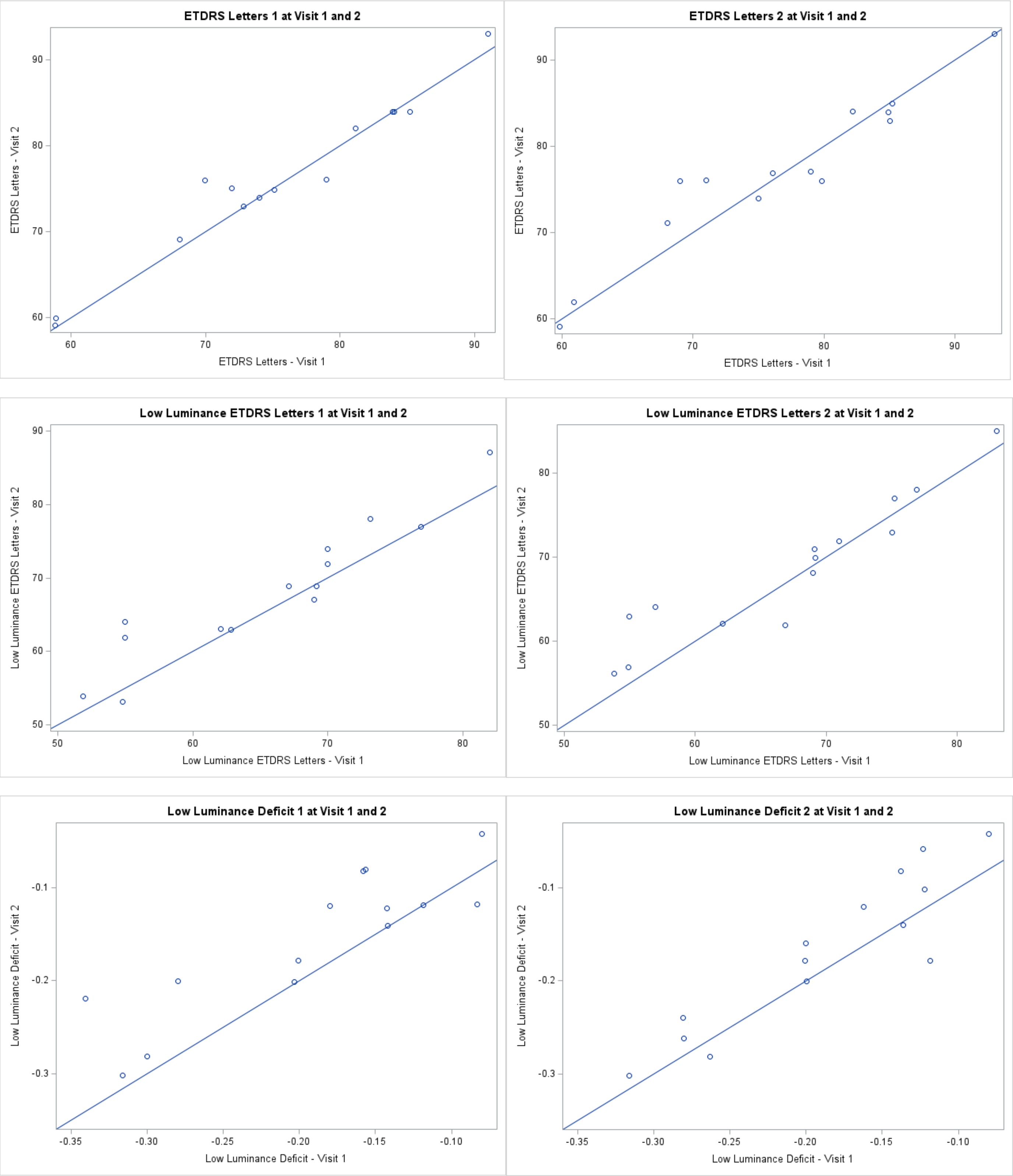
ETDRS Letters 1 at Visit 1 and 2
ETDRS Letters - Visit 2
ETDRS Letters - Visit 1
ETDRS Letters 2 at Visit 1 and 2
ETDRS Letters - Visit 2
ETDRS Letters - Visit 1
Low Luminance ETDRS Letters 1 at Visit 1 and 2
Low Luminance ETDRS Letters - Visit 2
Low Luminance ETDRS Letters - Visit 1
Low Luminance ETDRS Letters 2 at Visit 1 and 2
Low Luminance ETDRS Letters - Visit 2
Low Luminance ETDRS Letters - Visit 1
Low Luminance Deficit 1 at Visit 1 and 2
Low Luminance Deficit - Visit 2
Low Luminance Deficit - Visit 1
Low Luminance Deficit 2 at Visit 1 and 2
Low Luminance Deficit - Visit 2
Low Luminance Deficit - Visit 1

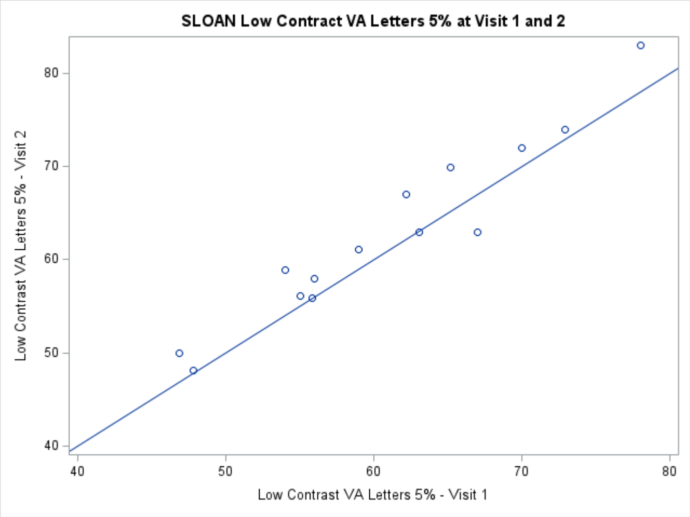
SLOAN Low Contract VA Letters 5% at Visit 1 and 2
Low Contrast VA Letters 5% - Visit 2
Low Contrast VA Letters 5% - Visit 1

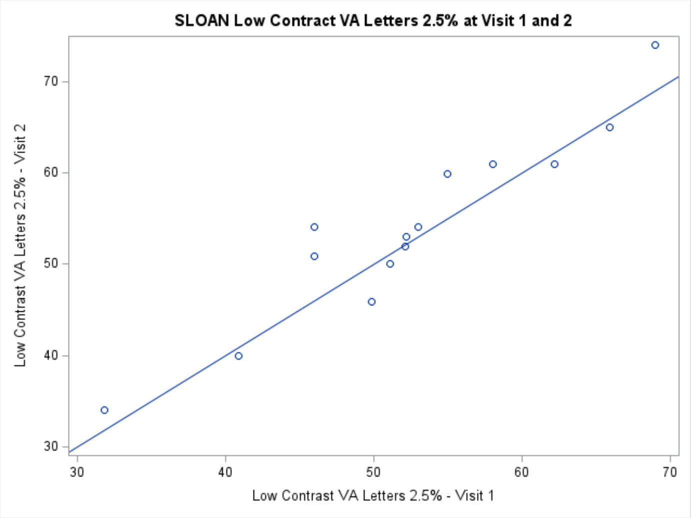
SLOAN Low Contract VA Letters 2.5% at Visit 1 and 2
Low Contrast VA Letters 2.5% - Visit 2
Low Contrast VA Letters 2.5% - Visit 1

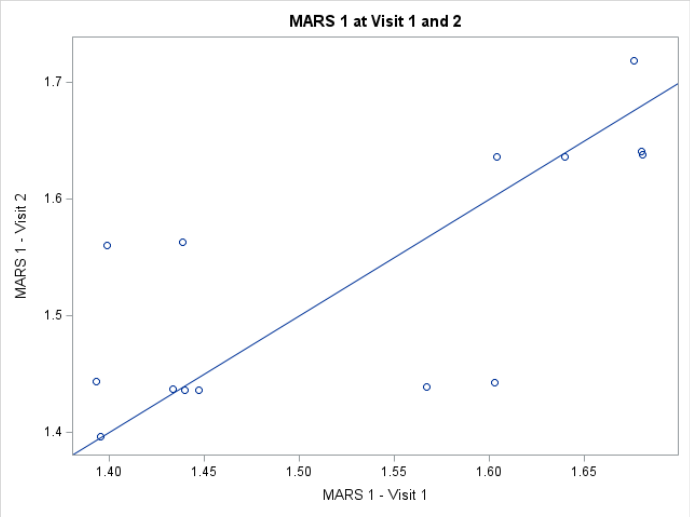
MARS 1 at Visit 1 and 2
MARS 1 - Visit 2
MARS 1 - Visit 1

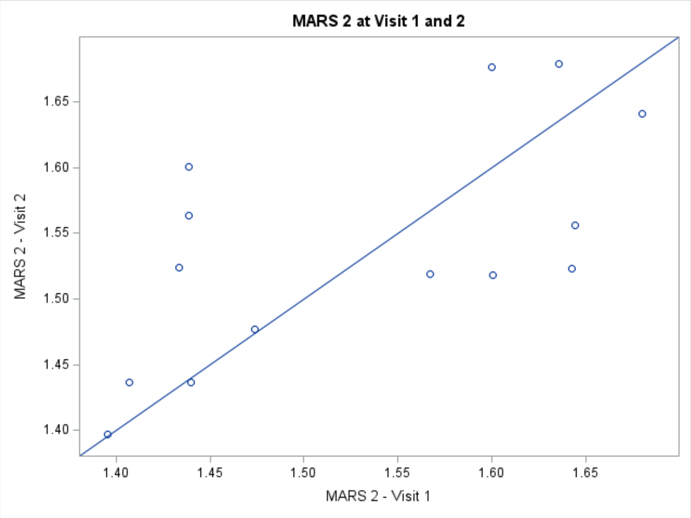
MARS 2 at Visit 1 and 2
MARS 2 - Visit 2
MARS 2 - Visit 1

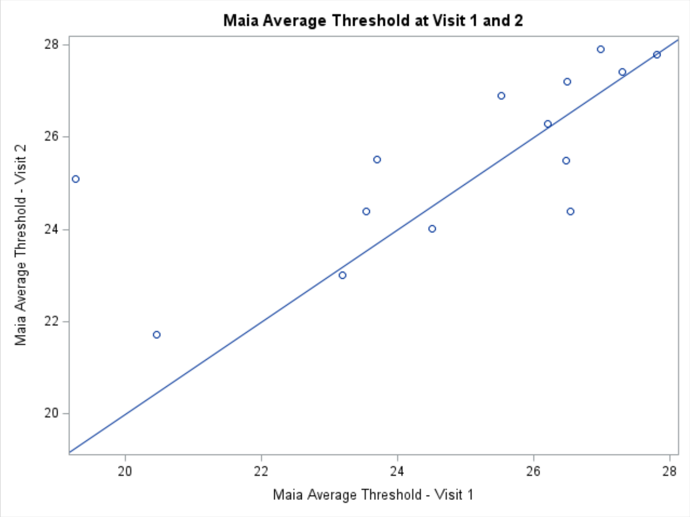
Maia Average Threshold at Visit 1 and 2
Maia Average Threshold - Visit 2
Maia Average Threshold - Visit 1

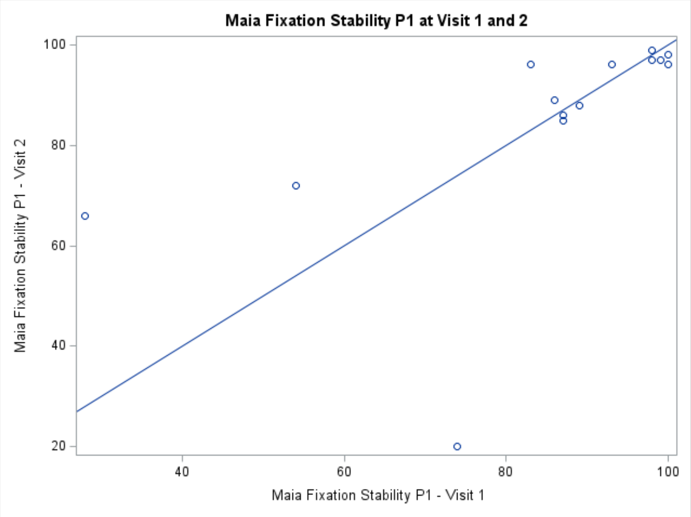
Maia Fixation Stability P1 at Visit 1 and 2
Maia Fixation Stability P1 - Visit 2
Maia Fixation Stability P1 - Visit 1

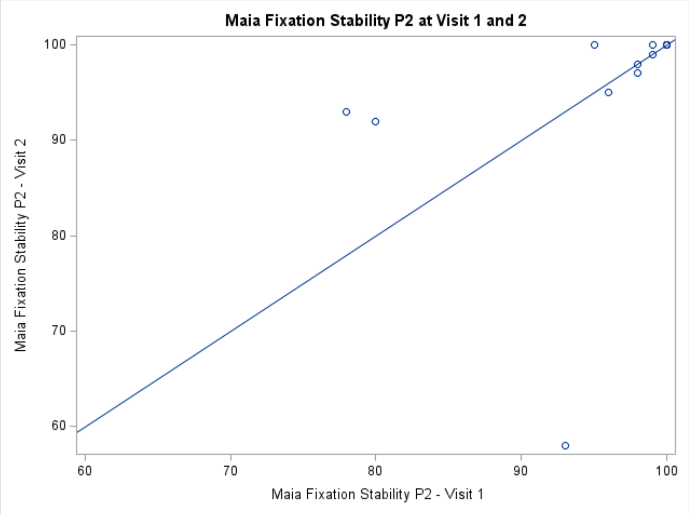
Maia Fixation Stability P2 at Visit 1 and 2
Maia Fixation Stability P2 - Visit 2
Maia Fixation Stability P2 - Visit 1
60
70
80
90
100

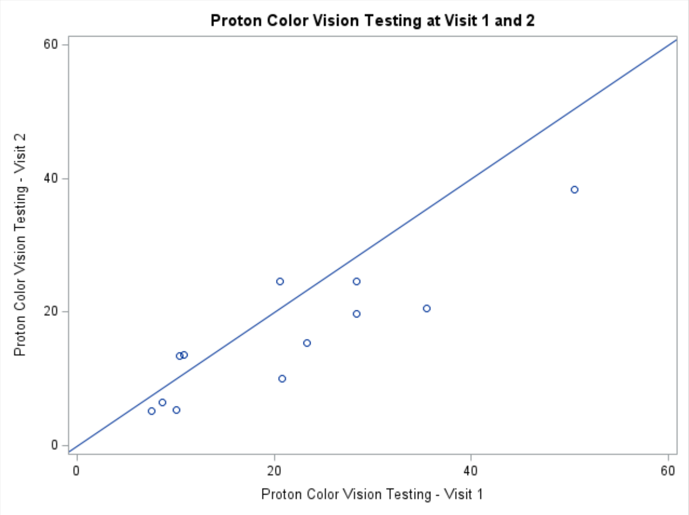
Proton Color Vision Testing at Visit 1 and 2
Proton Color Vision Testing - Visit 2
Proton Color Vision Testing - Visit 1
0
20
40
60

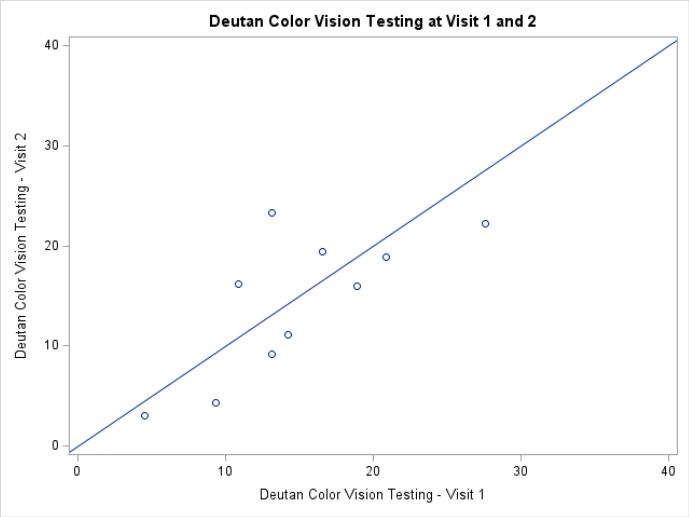
Deutan Color Vision Testing at Visit 1 and 2
Deutan Color Vision Testing - Visit 2
Deutan Color Vision Testing - Visit 1
0
10
20
30
40

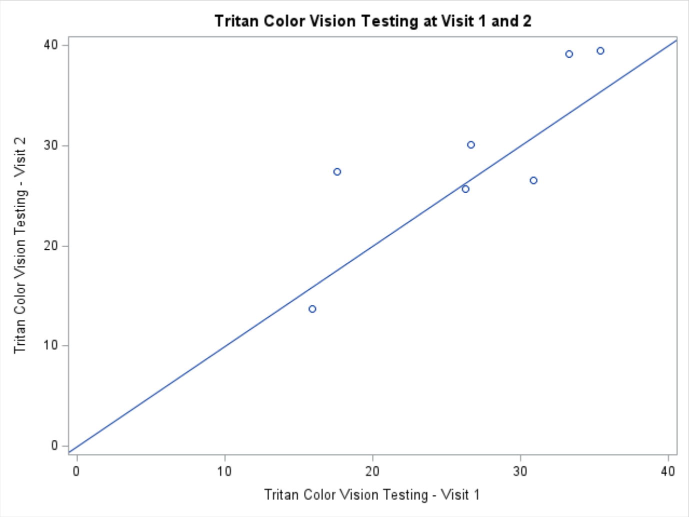
Tritan Color Vision Testing at Visit 1 and 2
Tritan Color Vision Testing - Visit 2
Tritan Color Vision Testing - Visit 1
0
10
20
30
40

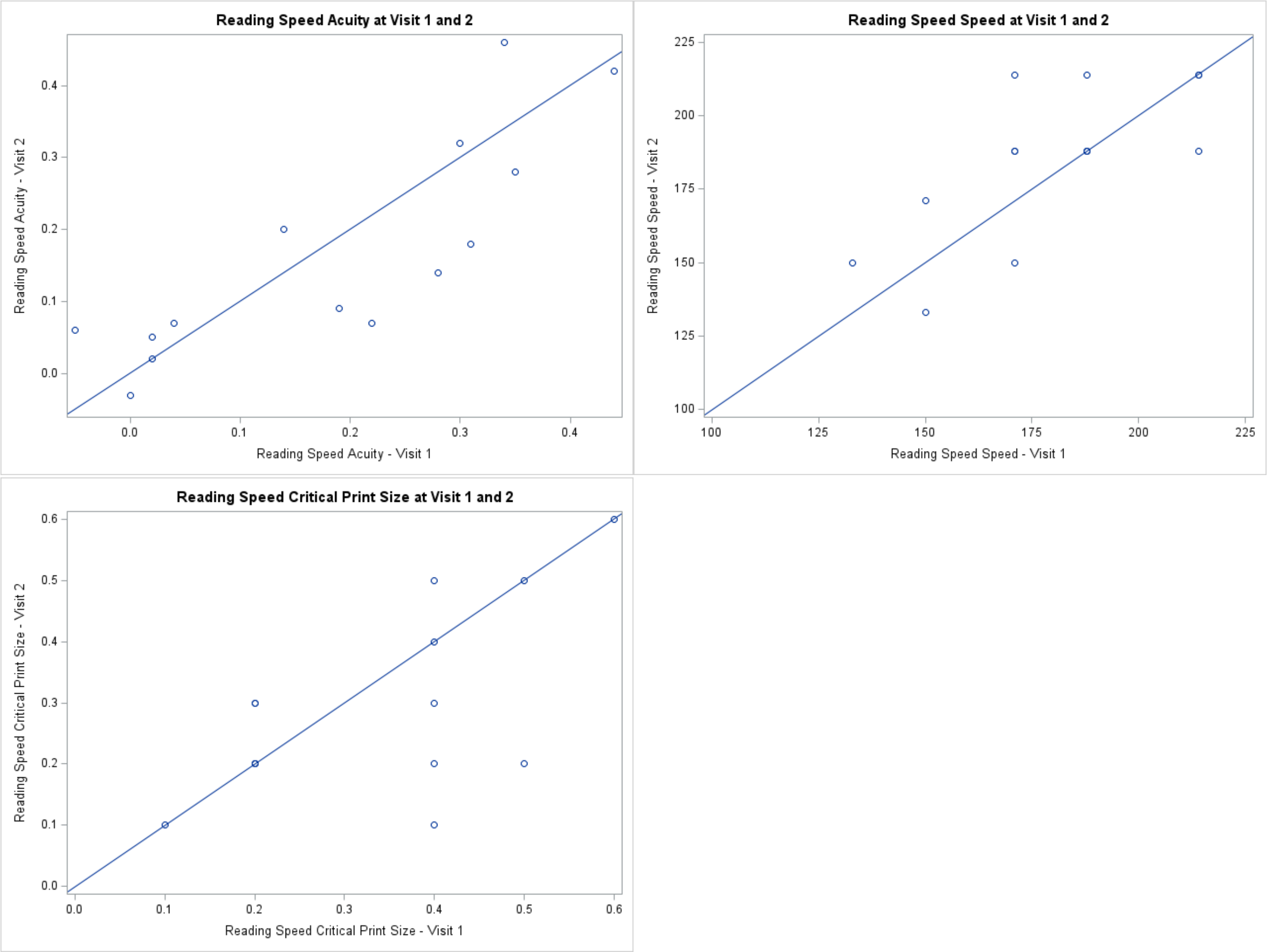

Supplemental Figure 1. Ocular measurements at visit 1 and visit 2 for the study eye. Each circle represents a patient's study eye. Visit 1 is on the x-axis and visit 2 on the y-axis. The blue line represents perfect agreement between the two visits.
